# A Distributed Big Data Analytics Architecture for Vehicle Sensor Data

**DOI:** 10.3390/s23010357

**Published:** 2022-12-29

**Authors:** Theodoros Alexakis, Nikolaos Peppes, Konstantinos Demestichas, Evgenia Adamopoulou

**Affiliations:** 1 School of Electrical and Computer Engineering, National Technical University of Athens, Zografou, 15773 Athens, Greece; 2 Department of Agricultural Economy and Development, Agricultural University of Athens, 15855 Athens, Greece

**Keywords:** big data, distributed architecture, sensors, machine learning

## Abstract

The unceasingly increasing needs for data acquisition, storage and analysis in transportation systems have led to the adoption of new technologies and methods in order to provide efficient and reliable solutions. Both highways and vehicles, nowadays, host a vast variety of sensors collecting different types of highly fluctuating data such as speed, acceleration, direction, and so on. From the vast volume and variety of these data emerges the need for the employment of big data techniques and analytics in the context of state-of-the-art intelligent transportation systems (ITS). Moreover, the scalability needs of fleet and traffic management systems point to the direction of designing and deploying distributed architecture solutions that can be expanded in order to avoid technological and/or technical entrapments. Based on the needs and gaps detected in the literature as well as the available technologies for data gathering, storage and analysis for ITS, the aim of this study is to provide a distributed architecture platform to address these deficiencies. The architectural design of the system proposed, engages big data frameworks and tools (e.g., NoSQL Mongo DB, Apache Hadoop, etc.) as well as analytics tools (e.g., Apache Spark). The main contribution of this study is the introduction of a holistic platform that can be used for the needs of the ITS domain offering continuous collection, storage and data analysis capabilities. To achieve that, different modules of state-of-the-art methods and tools were utilized and combined in a unified platform that supports the entire cycle of data acquisition, storage and analysis in a single point. This leads to a complete solution for ITS applications which lifts the limitations imposed in legacy and current systems by the vast amounts of rapidly changing data, while offering a reliable system for acquisition, storage as well as timely analysis and reporting capabilities of these data.

## 1. Introduction

### 1.1. Overview

The ever-increasing needs for sensor data retrieval, storage, processing and analysis impose the need to study, examine and implement distributed architectures for big data management systems. The rapid evolution of telecommunications technologies has paved the way for real-time interconnection and data transmission from and to heterogeneous applications and systems [1]. Such distributed architectures, in most cases, require different components, with various software and hardware requirements, to be deployed in order to perform the following processes as efficiently as possible: data transmission, data gathering, data storage, data analysis, and reporting.

In addition to big data technologies, data mining, machine learning (ML), data fusion, and social network methodologies are being adopted by a constantly increasing number of organizations and companies with great results [2]. Among the many applications and systems of big data technologies are the intelligent transportation systems (ITS) or the transportation domain in general [3]. 

ITS incorporate state-of-the-art technologies and tools, including advanced sensor technologies, data acquisition and transmission technologies as well as smart control and actuators, in order to enhance people and freight transportation solutions [4]. In this light, it is not strange that distributed architectures have attracted the attention of the ITS research community in recent years [5]. By designing a distributed architecture, different systems, serving different functionalities, can be separated into homogeneous groups. For example, the on-board vehicle data acquisition system hosts all sensors and in-vehicle communication protocols while the data storage system is differentiated in terms of functionality, from data acquisition and real time analytics systems. Even though these systems are separated and distributed across the entire architecture, all need to function independently but also in cooperation with one another. The harmonization and synchronized operation of the various components in a distributed solution is one of the main aims of the architecture and the applied system that is presented in this study.

The main inspiration and the driving force of this study was to create a common ground for different heterogeneous applications of an ITS. These applications might be connected to the vehicle itself, can run as a smartphone application used by the drivers or as a web-based application for the highway and the fleet administrator’s management as well as work with any other third-party application that could benefit from the services of the architecture proposed and the system designed and implemented in this study. In the next paragraph (Section 1.2. Related Works), numerous studies that exploit current technologies in order to add value to the ITS domain can be found, but in most the integration of these technologies into a common open and scalable platform is not in place. Considering this gap and by engaging different technologies, including both state-of-the-art solutions such as big data, ML and analytics as well as legacy solutions such as SQL databases and REST APIs, the architecture proposed and the platform implemented offers an “one-stop shop” for data management, processing and analysing in the ITS domain. The deployed system is future-proof and can also function in a plug and play manner regarding its interconnection to the currently existing infrastructure, especially of administrators of highways and fleets.

In this context, a distributed big data analytics (DBDA) platform is designed and implemented exploiting the advantages of big data, machine learning and cloud technologies. This DBDA platform utilizes the advantages of the aforementioned technologies in the following key aspects: (i) vast amounts of heterogeneous and complex data that are created by vehicles and roadside infrastructure can now be handled efficiently, (ii) the DBDA platform can improve the operation efficiency of the entire system by analysing current and historical massive vehicle and road data, and (iii) through big data analytics the safety level of the road and the drivers can be significantly improved. For this purpose, data collected from the vehicles are transmitted to the DBDA platform where, after appropriate processing and analysis, outlying conditions can be predicted and identified in order to warn the drivers and alarm operational management centers for accidents, closed lanes, etc. In addition, big data analytics make it possible to classify anonymous drivers’ behaviour using statistical methods and tools applied to collected driving data. This drivers’ profiling and traffic congestion conditions can assist in various relevant operations such as insurance calculations, etc.

The distributed big data analytics architecture and platform presented in this study creates a common working environment for all stakeholders of an ITS solution, enabling them to interact with the platform as both data consumers and contributors. Furthermore, from a communications viewpoint, it enables vehicle-to-vehicle (V2V) as well as vehicle-to-third party application (V2X) interactions both for real time as well as for historical data exchange. Through suitable data exchange and analysis, it is possible to reach useful conclusions and take informed actions towards enhanced road safety and efficiency as well as improved drivers’ behaviour.

The main objective of this study is to create a common framework, integrating all the technologies involved in the context of an ITS solution which will serve as an interjection of all data flows. Moreover, the need to cater to the increasing requirements of data availability and processing, led to the engagement of state-of-the-art tools and methods such as big data, ML and analytics in the proposed distributed architecture. It is worth mentioning that the feasibility of this solution was in question during the designing and implementation phase of this very system as many technological limitations had to be overcome. After almost three years of validation and testing of the proposed system, the results achieved are quite encouraging in terms of system availability and reliability. Thus, the contribution of this study is twofold: (i) first, to present and describe both the design and the implementation of the DBDA system and (ii) second, to prove the feasibility and effectiveness of such integrated solutions of different technologies, which until now have been tested separately but not as a whole, especially in operating environment conditions and during a long-time span as was the case in this study.

The remainder of the paper is organized as follows: first, the paper features related works of distributed systems and big data analytics technologies in the ITS domain, and then continues with a presentation of the proposed distributed big data analytics architecture. Subsequently, the variety of data formats hosted by the platform is presented, followed by a discussion on the platform’s analytics capabilities. Lastly, useful conclusions are drawn related to the DBDA platform’s design, development, and feasibility.

### 1.2. Related Works 

The vast volume of data originating from the growing number of sensors installed both on vehicles as well as on highways has attracted the attention of the research community which wishes to explore new methods and techniques in order to facilitate the constantly increasing needs for efficient and fast data handling. Data gathered from different sources in the ITS domain such as sensors, GPS, satellite traffic data and more create a heterogeneous ensemble of data that cannot be handled effectively by conventional tools and methods [6]. Nevertheless, the utilization of big data analytics can benefit ITS by providing the ability to efficiently handle vast amounts of heterogeneous and complex data and by enabling near real-time advanced analytics which can offer safer and more robust management in the ITS domain [7]. In this light, Kaffash et al. [8] performed a detailed review of big data algorithms and applications for ITS. In addition, Mohandu and Kubendiran [9] presented an extensive literature review on big data in ITS by describing different architectural approaches and applications. In their study, they provided an overview of various architectural schemes and, finally, adopted a similar scheme as the three-layer architecture proposed by Zhu et al. [7].

In reality, a large proportion of data in the ITS domain originated from the vehicles’ sensors. A summarization of the vehicle sensor data (VSD), as abbreviated by He et al., is presented in [10]. The goal of He et al. was to indicate the increasing production and availability of VSD as well as the rising need of big data technologies. In this light, Yoo et al. [11] presented a solution, which uses data coming from vehicle sensors and their environment in general, to assist in the operation of autonomous vehicles. In their study, they focus on autonomous vehicles that are directly connected to a big data platform. Yoo et al. utilize technologies like Hadoop [12], Apache Spark [13] and Kafka [14] and suggest a solution which can be scalable and efficient based on the attributes of these software components. Their distributed big data architecture solution seems to be feasible and adequate for their application. Yoo’s et al. [11] solution features similar ideas compared to our present study but it is focused on autonomous vehicles and was not tested in an operating environment for an adequate time-span, as the proposed platform did. In the same direction, Sengul et al. [15] performed a detailed depiction of different tools and frameworks needed to create a distributed big data-powered ITS. Their study mainly focused on the available frameworks and tools and how these can be divided and combined in order to create a common architecture. However, this study [15] only focuses on an architecture proposal without any relevant development, testing and evaluation. Digging further into the distributed data fusion architectures for vehicle sensor data, Tiedemann et al. [16] proposed a data management system as part of the Smart Adaptive Data Aggregation (SADA) project. Their solution considers a distributed architecture which involves smartphones, cars and backend infrastructure as a unified distributed system. Preliminary results of their study proved the feasibility of their proposed solution [16]. This solution relates to our own efforts since it engages various sensors and services that cooperate to create a unified framework. However, its results are preliminary, and the suggested solution was only evaluated in laboratory conditions and not in an operating environment. 

Even though techniques, methods and tools enabling the collection of vast volumes of data do exist, the samples collected, in most cases, are non-structured or semi structured, scarce and even sometimes corrupted. This is indeed a main challenge for most big data applications. To this end, Gohar et al. [17] proposed the SMART Transportations System Server (TSS) framework which, essentially, is a big data analytics platform consisting of four different units, namely: (i) the big data acquisition and preprocessing unit; (ii) the big data processing unit; (iii) the big data analytics unit and the (iv) data visualization unit. Their solution proved feasible and able to handle different heterogeneous data efficiently even in its first proof-of-concept deployment [17]. In the same direction, Babar and Arif [18] demonstrated an Internet-of-Things (IoT) big data analytics solution in an ITS environment. Their solution consists of three distinct stages, in particular: (i) big data organization and management; (ii) big data processing and analysis and (iii) big data service management. Each of these stages performs a different task which is essential for the next stage. More specifically, during the first stage, data acquisition and preprocessing are carried out which are followed by the stage of processing and analysis which feeds its results to the final decision making and reporting stage [18]. Moreover, Darwish and Abu Bakar [19] explore the potential of fog computing and big data analytics in ITS applications. Their solution adopts the three-layer architecture of most fog computing applications and adds one extra layer in order to facilitate the specific needs of the transportation domain. Their experiments reveal that an efficient big data analytics architecture for the ITS domain should combine different components in order to best exploit the advantages of each. More specifically, fog computing can be used for real-time analytics but its capabilities, as the needs of data processing increase, are limited. On the other hand, cloud computing can overcome this limitation, but in terms of real-time response performance, latency-sensitive applications could suffer delays [19]. Thus, after proper experimentation, Darwish and Abu Bakar concluded in a solution which demonstrates a three-dimension architecture in order to enable real-time big data analytics in an Internet of Vehicles (IoV) environment. These three dimensions involve intelligent computing, real-time big data analytics and the IoV [19]. Considering the approaches in [18,19], the data structure and real-time analysis were some of the first considerations of our suggested solution. Thus, as presented later in our study, the preprocessing stages of data are crucial while real-time monitoring and analytics were enabled by the development of a hybrid database system of SQL and NoSQL databases. 

Torre-Bastida et al. [20] performed an extensive literature review on the use of big data in the transportation sector, and more specifically, on the associated trends and challenges. In this study, the authors classify big data analytics into three categories: descriptive, predictive and prescriptive. In addition, among the challenges identified, the feasibility of real-time analytics, the security and privacy issues as well as the availability and the heterogeneity of data are included [20]. Another interesting study was conducted by Montoya-Torres et al. [21] who studied the adoption of big data analytics in the ITS domain. Their findings indicated that most of the efforts examined were focused on traffic flow prediction, safety, vehicle and/or driver behaviour as well as route evaluation. In addition to related studies, Montoya-Torres et al. [21] proposed a high-level abstract architecture concerning data analytics for ITS. Their architecture consists of five different service layers, namely: (i) the decision-aid layer; (ii) the visualization layer; (iii) the data warehousing layer; (iv) the data bunker layer and the (v) data normalization layer [21]. As obvious from the above, several interesting studies and suggested architectural schemes can be found in the literature. However, the majority are limited to being conceptual approaches that have not been fully, or sometimes even partially, implemented and have thus not yet been evaluated for their feasibility in operating conditions as the approached proposed in this study. 

Big data analytics in ITS can provide useful insights on the status and the conditions of a transportation system. Therefore, many studies focus on the prediction of traffic and road conditions using different methods such as Artificial Neural Networks (ANN) [22,23], Long-Short Term Memory (LSTM) Neural Networks [24], Bayesian networks [25], and deep learning [26,27] approaches and methods relying on an ensemble of different single predictors of traffic [28]. Other interesting approaches focus on warning systems, such as the one proposed by Teke and Duran [29] or dangerous driving events modelling platforms, such as the one proposed by Alvarez-Coello et al. [30]. More specifically, Alvarez-Coello et al. engaged the Random Forest (RF) algorithm as well as a Recurrent Neural Network (RNN) in the design of their platform. In addition, Liu et al. proposed a complete solution which foresees the exchange of data among a central platform and a highway system in China [31]. Following the same path, An and Wu [32] proposed a big data assisted framework for traffic prediction. Their solution consisted of two main pillars, i.e., the prediction module and the data forwarding module of the prediction results. The Temporal Convolutional Network (TCN) in their case outperformed RNN and LSTM methods by achieving better prediction accuracy results. Yang et al. [33] proposed a Traffic Analysis Zone (TAZ) solution based on multiple data sources. Their study engages the Fuzzy C-Means algorithm for clustering the TAZs. Their contribution involved big data to handle the heterogeneous sources, similar to our proposed solution, but had some limitations such as the lack of data from private vehicles as well as time mismatches of incomplete data [33]. In a different direction, Mouammine et al. [34] chose to utilize social media posts in order to detect or predict possible road events (crashes, closed lanes, delays, etc.). Their approach adds a new perspective and a future direction that can be also hosted in our proposed DBDA platform presented in Section 2. Nguyen et al. [35] in a recent study, presented the role of big data analytics in ITS and provided an overview of how big data can help smart cities in addition to the transportation domain. Their main conclusion was that the collaboration among different stakeholders, technologies, tools and authorities is crucial in order to develop such systems. This is also one of the main points of the present study as the holistic approach envisaged, developed, and presented herein aims to host and serve different stakeholders, applications and services.

The study of related works as well as the expanding interest on distributed architectures, big data and cloud services led us to explore and design a holistic solution which combines these technologies in a common architectural framework. Our proposed DBDA platform, presented in detail in Section 2, is divided into three layers, namely the data exchange and collection layer, the data processing, analysis and cloud layer and the monitoring-application layer. The three layer architectural scheme, adopted by other studies (e.g., [7,9,36]) as well, was selected as it efficiently materializes the notion of conceptualization in such systems. Considering the study of related works, the main limitations identified included the design only at the conceptual level of the suggested solutions without adequate validation and testing, the over-focusing on specific tasks such as traffic prediction, the constraints due to lack of high-quality data and the non-validation in real-life operating environments or for a time duration adequate to support their feasibility, efficiency and performance. 

## 2. Distributed Big Data Analytics Architecture

**Figure 1:**
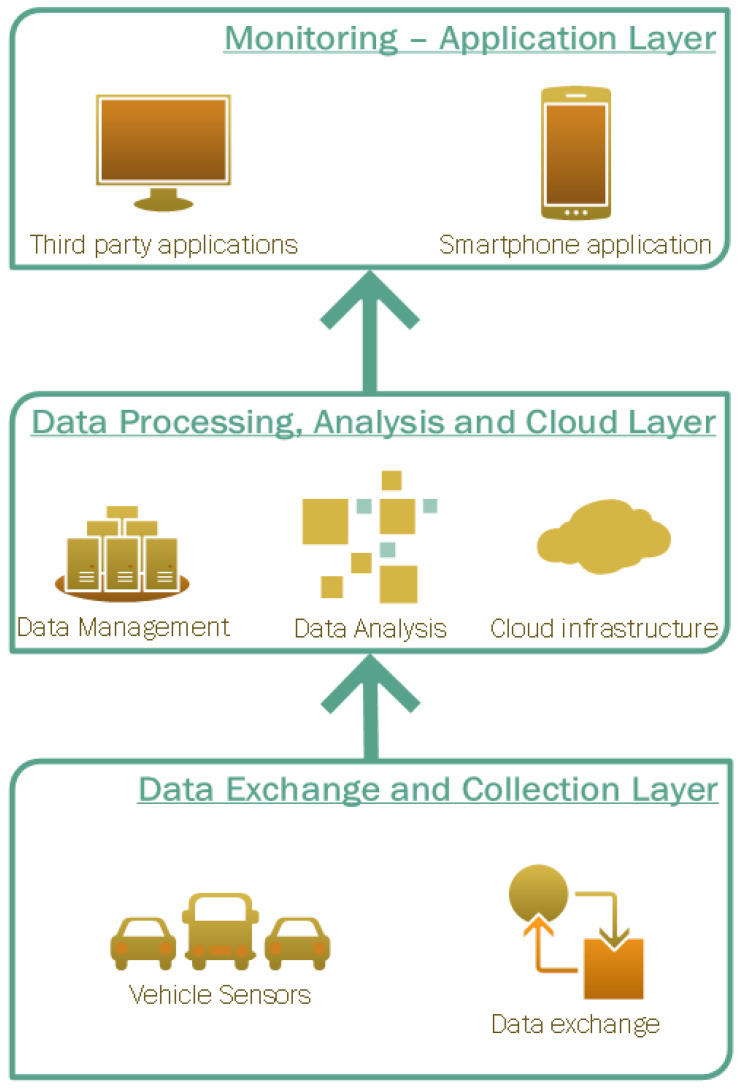
The 3-layers of the DBDA architecture.

The distributed big data analytics platform designed and developed for the purposes of this study is comprised of three distinct layers as shown in Figure 1.

**Figure 2:**
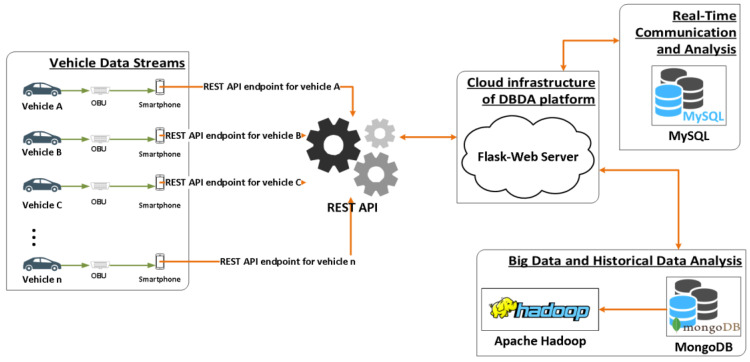
REST API for vehicle sensor data flow.

In a bottom-up approach, the “data exchange and collection layer” hosts all the infrastructure necessary to gather and store data originating from vehicle sensors. More specifically, this layer contains all the interfaces required to retrieve data from the vehicles’ sensors and transmit them to the DBDA platform for further processing. The information flow from the vehicles to the DBDA platform is depicted in Figure 2.

Moreover, the “data exchange and collection layer” was designed to employ advanced data collection techniques for monitoring and collecting multiple types of data formats (structured and semi-structured) from various sources (vehicles, monitors and users).

**Figure 3:**
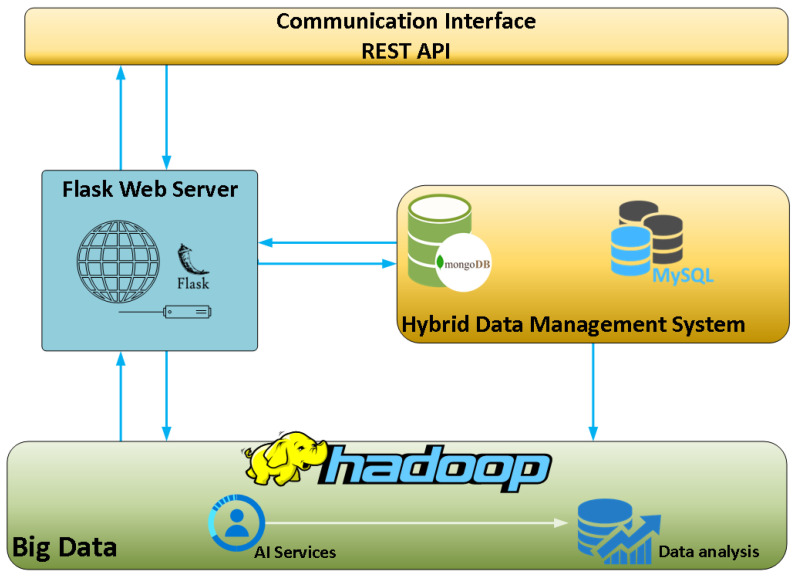
Layer two components of the DBDA platform.

Moving up to the next layer, one can clearly identify the three pillars of the DBDA platform as this layer hosts and integrates the cloud infrastructure, the big data management systems as well as the data preprocessing and analysis modules. The core infrastructure of the DBDA platform is depicted in Figure 3.

As seen in Figure 3, the DBDA platform comprises several different open-source tools and technologies. The “data processing, analysis and cloud layer” applies feature engineering techniques to preprocess the collected raw data and extract hidden information using descriptive and predictive analysis algorithms. More specifically, the components used and depicted in Figure 3, mentioned in a bottom-up order, are:

- The big data management tool. The Hadoop framework is engaged for big data management, alongside a data analysis and reporting component which utilizes artificial intelligence (AI) services. This data analysis and reporting tool provides timely analyses of the data based on pre-trained models, as well as periodical reports and near-real-time alerts/messages to vehicles or external applications.
- The hybrid database system. This component consists of a NoSQL database, in this case, the well-known MongoDB, which caters to the big data storage needs, as well as a SQL database which aids in addressing the real-time requirements by retrieving, streaming and storing small amounts of data. The choice of using a hybrid implementation of both SQL and NoSQL databases was made by taking into consideration the requirements for both real-time analysis as well as storage of vast volumes of data which are rapidly generated.
- The Flask-web server. Flask is a Pythonic microweb framework which renders the process of web application design and deployment much easier by incorporating the client–server architecture which is essential for such applications as it enables data transfer between the client(s) and the server. This component, together with the communication interfaces (REST-APIs) which are described next, practically enables the cloud infrastructure of the DBDA.
- Communication interfaces—REST APIs. The communication between the platform and the vehicles is achieved using REST APIs which are provided by the Flask microweb framework described above. This development setup enables two-way communication from and to the platform, as well as from and to the vehicles and external applications. Specifically, the vehicles’ data are transferred through REST APIs, after the specification and use of the appropriate data format. In a continuous session, requests are sent from the vehicles to the platform’s server, using a particular URL and an endpoint together with the data in a specific format. In this way, the API is able to apply all possible CRUD (Create Read Update Delete) methods by using the appropriate query in order to achieve a specific operation. Moreover, a REST API interface is used to establish communication between third party systems and the platform in order to exchange data. Each application can retrieve data either through the relational database for real time analysis or through the NoSQL database for batch data (historical) analysis. REST is a type of software protocol and service that enables communication between different software applications or systems. The selection of the specific API was based on the following aspects: (i) the high popularity of this API architecture, (ii) the compatibility of REST with a variety of messaging formats (e.g., JSON, YAML, XML), (iii) the higher performance of servers and the faster networks’ speed, (iv) the abstraction of technical details and (v) the simplification of the development procedures.

**Figure 4:**
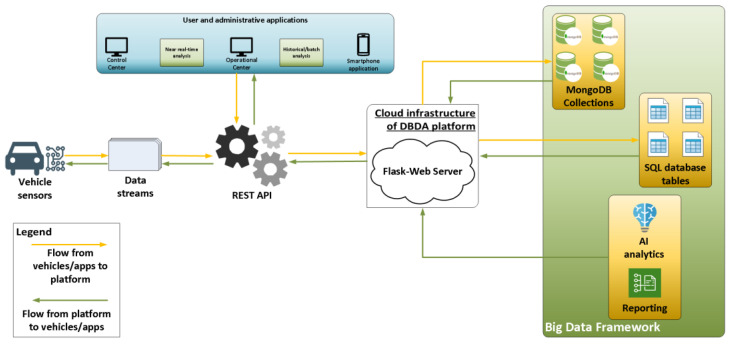
Data flow and exchange among applications, sensor vehicles and the DBDA platform.

The third and upper layer of the proposed architecture caters to the data exchange from and to the smartphone applications of drivers or administrators as well as third party applications such as the control centers of highways and fleets. The “monitoring—application’ layer” leverages the analysis results of the previous layer to profile the driving behaviour, provide the corresponding (driving behaviour) label to each driver as well as process other related information such as traffic conditions and statistics for vehicles and drivers that are useful for reporting functionalities. All the end-users’ applications which exploit the results of the DBDA platform, such as warning, reporting, messaging systems and more, reside in this layer. A schematic overview of the DBDA platform and the data flow among the different components, applications and vehicles is shown in Figure 4.

As is obvious from Figure 4, all three layers presented before are linked to one another and create a common framework consisting of distributed components of sensors, big data technologies, analytics and cloud infrastructure.

The credibility of the functions of the DBDA platform is ensured by maximizing the scalability benefits of the cloud using NoSQL architecture, leveraging the speed of both DBs (for different purposes) that provide high availability and auto-replication features, satisfying the requirement for ACID (Atomicity, Consistency, Isolation, Durability) [37] compliance and highlighting the capability of connecting heterogeneous types of data sources to be analyzed and presented by the system, as a result of the high schema flexibility.

During the design and the development phase of this platform, several risks and limitations were considered. First, the demand for real-time analytics and reporting imposed the need for advanced pre-trained machine-learning models and procedures. This led to the need for expensive hardware featuring state-of-the-art specifications, such as high-end processors, plenty of gigabytes or RAM, etc. The state-of-the-art technologies as well as the variety of functionalities hosted in the DBDA platform require sophisticated software development due to the inherent high complexity. Moreover, the cloud infrastructure increases the demand for hardware resources in order to ensure redundancy and high availability of the platform. The cloud-based nature of the platform highly relies on the availability of internet connection of the different components. As presented in [38], data quality actions are performed to ensure proper functionality of the entire system as well as the credibility of analytics.

Another aspect of the distributed cloud architectures that was considered during the design and development of the proposed platform is security and privacy of both the data and the end-users. Thus, cryptography techniques (e.g., digital signatures) were put in place in order to avoid breach attacks and data leakage as well as to ensure non-repudiation. In addition, in the same direction, authentication, auditing and logging services are integrated in the entire infrastructure. In this light, the access to obtain and retrieve data through the REST API offered is feasible only for accredited users and applications who are authenticated via their respective credentials. In this way, the credibility of data coming from vehicle sensors cannot be denied, ensuring non-repudiation. Moreover, the entire cloud infrastructure and the REST APIs integrate SSL functionality and appropriately configured firewalls in order to avoid connections of unknown origin. Monthly scheduled updates are also performed on every machine in order to assure that the latest security updates are applied.

## 3. Data Formats of the DBDA Platform

The DBDA platform was designed to be data agnostic in order to support different data formats from different vehicle sensors and/or third-party applications. During the platform’s performance evaluation phase, the data hosted in the platform consisted of data originating from two different vehicle fleets as well as from a major road network operator. Table 1 features an overview of the data structure of the different data sources.

As seen in Table 1, several diverse types of data and formats originating from different sources coexist in the DBDA platform without any restrictions. This validates the fact that the platform is data agnostic and can host various heterogeneous data formats efficiently, which is a necessary ability for applications where functionality is distributed to different systems in order to compose a holistic solution.

In addition to the data structures provided as input to the DBDA platform, other data generated by the analytics modules are combined to the input data and become available to the third layer’s modules and especially to the monitoring and control applications. These data structures were decided in collaboration with the platform’s end users in order to provide the most useful insight to the actual platform users. This information is produced using the analytic capabilities of the DBDA platform which are described later in this study. More specifically, Table 2 presents some indicative data structures produced and supported by the platform.

It is worth noting that the above data was anonymous, for privacy reasons, and the driver associations were made only with an identification number and not with personal information such as name and surname. The intelligent analysis services described in the next section are performed on the seamless data flow from and to the DBDA platform both from the vehicles and from third party applications. This is achieved through the communication interfaces and, more specifically, the dedicated REST APIs that are available to authorized users of the platform.

## 4. Analytics and Statistics Produced by the DBDA Platform

It is noteworthy that due to the vast amount sent to and from the DBDA platform (by the time of writing this article over 20 million records were registered), it was deemed necessary to implement data quality control actions to ensure their correctness and suitability for analysis and identification of suitable statistics metrics. More specifically the following techniques were applied on the data set [38]:

- Range control
- Outlier check
- Sudden fluctuation check
- Duplication check
- Sequential check

The quality control procedures, implemented on the considered dataset, revealed hidden rules and useful patterns which could lead to applicable reasoning techniques. More specifically, the application of quality control techniques in the vast amount of involved data streams indicated potential problems that are directly connected with data analysis. Incorrect training processes of the developed machine-learning models and thus the deduction of erroneous conclusions could be some. 

The analytics of the DBDA platform were mainly focused on three pillars: (i) the detection of the outlying driver’s behaviour in terms of environmental aspects [39,40]; (ii) the classification of drivers and the evaluation of traffic conditions and (iii) the statistical analysis of attributes related to drivers and vehicles.

### 4.1. Outlying Environmental Driver’s Behaviour

**Figure 5:**
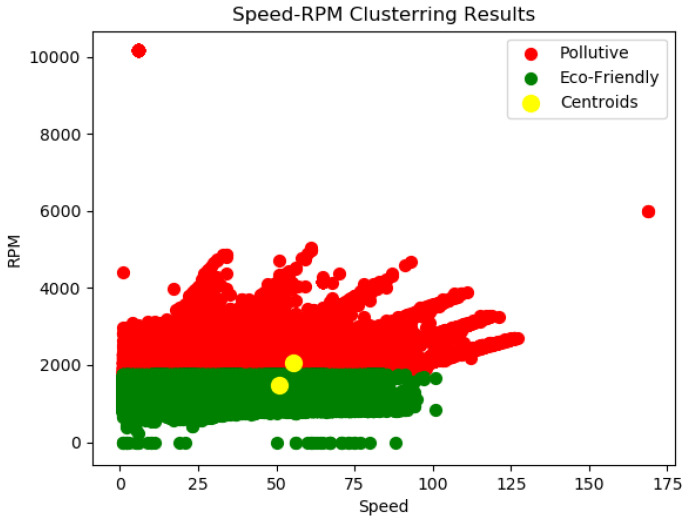
Clustering algorithm implementation for streams from car sensors [40].

Following the data quality control procedures and after the necessary automated preprocessing to transform the raw input data into a format suitable for further analysis procedures, a clustering methodology is followed in order to analyse historical batches of data contained in the NoSQL database. As described in [40], through the application, in the appropriate plot, of the elbow method [41] in order to determine the optimal number of clusters and the application of the developed K-means -based model [42] in order to discover the number of different groups which have not been explicitly detected and labeled in the data used, two classes of environmental driving behaviour profiles were identified. More specifically, the behaviour profiles which were characterized by the eco-friendly driving style and those for which their behaviour can be characterized as aggressive or pollutive as shown in the RPM-speed diagram depicted in Figure 5. 

Based on the methodology presented in [40], the labelling process utilized a series of metrics in order to cluster the data while an extended evaluation process was applied afterwards in order to indicate the method that performed best among the machine (ML) and deep (DL) learning algorithms considered. More specifically, the ML algorithms that were initially selected and tested were the Logistic Regression (LR), the Support Vector Classifier (SVC) and the Random Forest (RF) whilst the DL algorithms included the Multi-Layer Perceptron (MLP) and the Recurrent Neural Network—Long Short-Term Memory (LSTM). The selection of the aforementioned ML algorithms was based on the interpretability of the target output, the size of the training data as well as on the required training time evaluations. Based on the results presented in [40], the algorithm that performed best both in term of loss and accuracy as well as on execution time was the SVC algorithm. Thus, this algorithm was selected to train the model in order to produce near real-time labelling of the driver’s behaviour. In this way, the DBDA platform provided to interested users the results as described in Table 2, and more specifically in the rows describing the driver’s behaviour labelling data.

### 4.2. Clustering of Vehicle Data and Traffic Conditions Characterization

**Figure 6:**
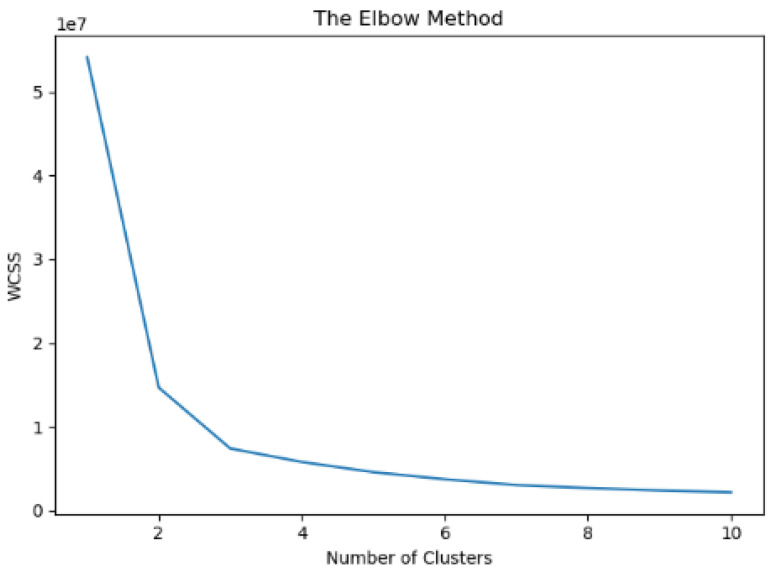
Elbow method result for clustering purposes of batch data of fleet 2.

The current section presents the results obtained from the cluster analysis (clustering) on data that originated from the set of raw data retrieved from eight different properly equipped vehicles of fleet 2. As can be seen in Table 2, those vehicles are equipped with a variety of sensors and provide diverse types of data. The clustering procedure engaged a dataset of approximately fifty thousand diverse records in terms of speed, acceleration, location and vehicle identifier, which were then used as input to the K-means algorithm (clustering) for training purposes. The elbow method was engaged in order to classify the data points into a number of consequent clusters. Thus, following the elbow method, the optimal number of clusters identified in this case was three (K = 3) as depicted in Figure 6. More specifically, the value at which the distortion of the variable related to the number of clusters starts to decline in a linear fashion is called “elbow”. This value is the number of the optimal clusters as defined by the elbow method. In our case, this number equals three, the point at which we stopped dividing the data into further clusters.

The choice of the K-means algorithm for clustering based on feature similarity was performed because it is one of the most widely used machine learning methods for unsupervised learning. More specifically, K-means aims to determine the number of similar object or behaviour groups that are more related to each other than to objects of other groups from a dataset of unlabeled input data. The K-means algorithm is commonly indicated as a prototype-based clustering algorithm, where each cluster is depicted by a prototype, which can be either the average of similar points (centroids) or the most frequently occurring points (medoid). In our case, the representation of each cluster is denoted by the Euclidean distances between the centroids and the existing vehicles’ data points. The squared Euclidean distance is a widespread method approach for calculating the similarity instead of the distance between the data objects and is calculated based on Equation (1) [43]:

(1) $$d\left(i,j\right)={\left(x_{i1}-x_{j1}\right)}^{2}+{\left(x_{i2}-x_{j2}\right)}^{2}+\ldots +{\left(x_{in}-x_{jn}\right)}^{2}$$

Based on the aforesaid, the K-means algorithm could be described as an optimization approach for minimizing the inside cluster Sum of Squared Errors (SSE), known as cluster inertia. The equation for the SSE is shown below (Equation (2)) where $\mu^{\left(j\right)}$ is the centroid for cluster *j* [44]:

(2) $$SSE=\sum_{i=1}^{n}\sum_{j=1}^{n}w^{\left(i,j\right)}\|x^{\left(i\right)}-\mu^{\left(j\right)}{\|}_{2}^{2}$$

Regarding our vehicle dataset analysis process in conjunction with the implementation of the described K-means algorithm, we investigated the existence of new possible correlations between the eight different vehicles in terms of the “speed” and “speed difference” attributes during discrete time moments, which were extracted from our vehicles’ dataset. Under these circumstances, we set the number of clusters K = 3, as was previously substantiated, and the maximum number of iterations equal to three hundred while we defined the algorithm to run fifteen times with different random centroids in order to select the one with the lowest SSE. In addition to the above configurations, a tolerance threshold equal to 1 × 10^−4^ (0.0001) was selected for detecting possible convergences regarding changes in the Within-Cluster Sum of Square (WCSS).

**Figure 7:**
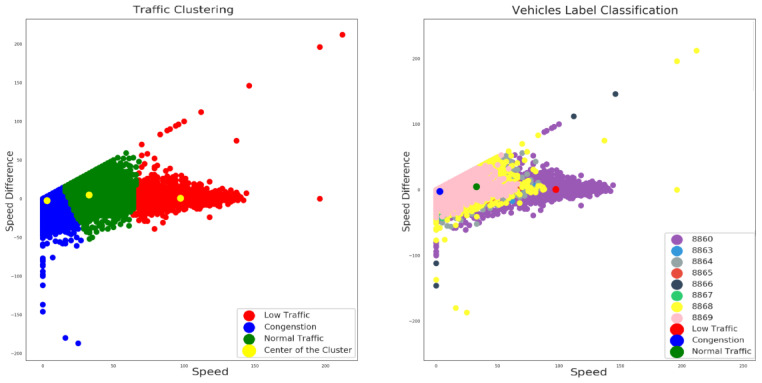
Traffic condition (**left**) and vehicles’ data distribution (**right**).

Consequently, the outcome of the application of the K-means algorithm to our vehicles’ dataset input is illustrated in Figure 7.

**Figure 8:**
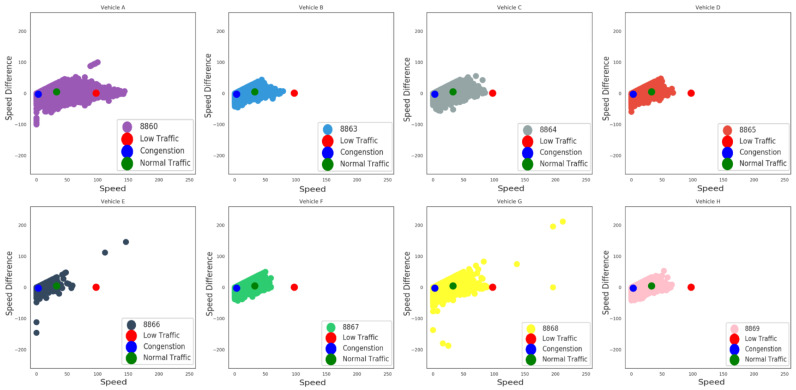
Data samples of each vehicle connected with the clustering centroids.

On the left side of Figure 7 the result of the clustering process is depicted where three different recognizable data group clusters were identified. The centroids (or the average centers) of each of the three clusters are illustrated with yellow marked labels. The identified clusters are considered to correspond to three different possible traffic conditions: (i) the blue color depicts a possible condition of congestion as a combination of low speed and negative speed differentiation values range; indicating that, due to the specific traffic situation, decelerations often occur; (ii) the green color indicates the combination of the medium speed values range and a mixed sample of speed differentiations that suggests normal traffic conditions; and eventually (iii) low traffic conditions are depicted with red color as a result of high speed values and speed differentiations close to zero values, implicating more steady and easy driving conditions. In addition, on the right side of Figure 7 another viewpoint of the described analysis with different label classification of the eight distinct vehicles ID, in terms of the current clustering results, is depicted. Figure 8 presents the samples taken for each of the eight vehicles alongside with the centroids of the clusters. It is obvious that most of the drivers usually “move” between the blue and the green centroid, a fact which implies that in most cases there is a certain degree of traffic. More specifically, vehicle A features the most samples on the third (red) cluster compared to the other vehicles. This fact indicates that the driver of vehicle A drives at high speed in low traffic conditions. In addition, drivers of vehicle E and vehicle G provide some extreme negative or positive measurements compared to most of the records of this specific dataset. These measurements show either a fault on the measuring equipment or some deviant and dangerous driving behaviour which must be investigated further in order to accurately identify the real cause.

The clustering procedure and the analysis performed by the DBDA platform on the batch data considered, highlight the platform’s capabilities to retrieve and analyze large batches of historical data in order to provide useful insights to fleet managers, highway administrators and drivers. 

### 4.3. Statistics and Driving Behaviour Patterns Extraction

Another useful ability of the DBDA platform is the reporting functionality of the analytics component. This functionality produces statistics both for vehicles and drivers (when the appropriate information such as shifts is available) and indicates possible driving behaviour patterns. 

In order to provide timely and accurate statistics in an efficient and trustworthy way, it is necessary to perform feature selection or reduction to the available attributes presented and described in Section 3. Driving behaviour feature extraction is done following the pre-processing stage of the raw data gathered, while the selection of certain features aims to maintain the information that allows the easiest separation/distinguishing of the various driving patterns. To this end, the reduction of the size of the data is desired so that their classification is faster and more efficient. The feature selection process maintained selected features from the data produced by vehicle sensors and these data were combined with the shifts data in order to bind the analysis results with the profile of each driver in anonymous way. The result of this process is the creation of new complex features, which are formed as a function of the combination of the original attributes, based on a specific, common key field. This common key field of the vehicle data and the shifts data is the timestamp attribute. The new set of data that is created includes a series of characteristics useful for extracting driving characteristics and statistics both for vehicles and drivers. The steps taken to complete the merge proceedings and extract report statistics include:

1. Feature selection from data collected by sensors and stored in the DBDA platform. In our case, the following features from Table 1 were selected: carPlate, fuelLevel, relThrottle, rpm, speedGps and timestamp.
2. Creation of subsets from the original data stored in the DBDA platform, e.g., a different subset for each vehicle carPlate.
3. Export and preprocessing of the shifts’ data.
4. Processing of the timestamps data so that they become of common format, and more specifically in the timedelta format [45].
5. Correlation of the shifts’ timestamps and the data from vehicle sensors. When the carPlate value is common for the vehicle and the shifts data and the timestamp of the vehicle sensor data is in the range of the start and the end of a shift timestamp limits, then the attributes selected in step 1 are merged with the corresponding shifts.
6. Finally, the dataset that was derived from the previous steps contains the following attributes/values:
  - carplate,
  - carplate_shift,
  - cartimestamp,
  - convertedCartimestamp,
  - speedgps,
  - rpm,
  - relthrottle,
  - fuelevel,
  - passengerID,
  - shiftname,
  - km_start,
  - km_stop,
  - timefrom,
  - timeto

### 4.4. Visualization and General Discussion of the DBDA Platform’s Results 

**Figure 9:**
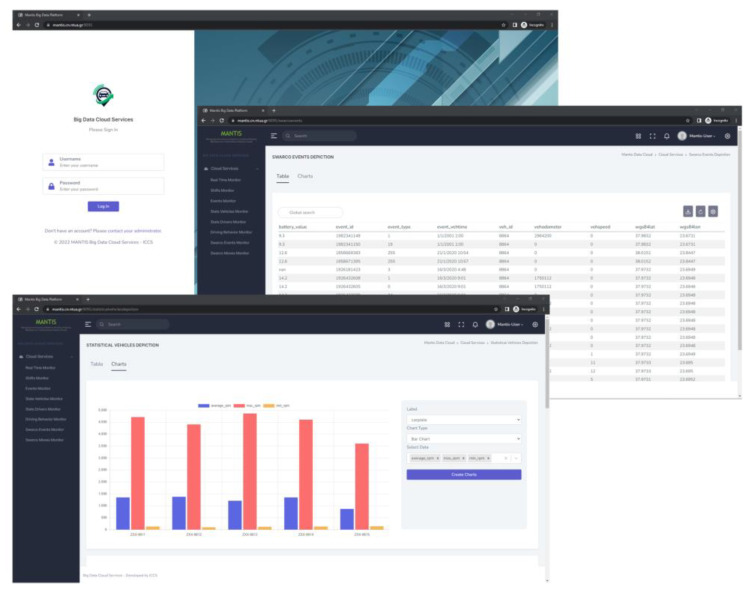
GUI of the DBDA platform.

The procedures described in the previous paragraphs of the current section enable the DBDA platform’s reporting tool to produce a plethora of statistics and reports that can be easily utilized by administrators and drivers, as they are available in the format presented in Table 2 (vehicle statistics and drivers’ statistics). Moreover, these statistics as well as the events and real-time information can be accessed through the Graphical User Interface (GUI) of the platform and any other third-party application that is authorized to have access to the communication interfaces. Some indicative screens of the platform’s GUI can be seen in Figure 9.

The present section demonstrates that the production of different formats of results and statistics, according to the end-users’ needs and requirements, calls for the application of a variety of methods and tools. Several different tools, frameworks and programming libraries were used, from the very first step of the data quality check to the final production of statistics reports, in order to offer a wide overview of results to interested stakeholders. As already discussed in Section 1.2, most of the relevant research efforts and literature studied for the purposes of this paper did not present unified solutions that produce robust and flexible insights on the results but rather focus on specific aspects of ITS such as traffic conditions. Furthermore, the value and the usefulness as well as the quality of results was not only validated in laboratory conditions, based solely on training data but was also approved by end-users, i.e., highway and fleet administrators who conducted a thorough inspection and evaluation.

## 5. Conclusions

The aim of the current work is to demonstrate the architecture of an innovative distributed big data analytics platform in the domain of ITS. The platform is running in an operational environment and is interconnected with a plethora of different vehicles and third-party applications, while its operation was validated over a period of three years. Through its continuous development and upgrade processes, it is expected that certain limitations of current intelligent transportation systems are addressed. 

In the context of this study, the high-level architecture of the suggested platform and its subcomponents were presented. Moreover, the data supported by the platform coupled with the platform’s analytics and reporting capabilities were discussed in detail, highlighting its support to administrative services as well as to the improvement of road and driving performance. Moreover, the scalability of the platform as well as its ability to accommodate a greater variety of analysis results as well as more multivariate types of data formats from different vehicles or even from different domains or sources was supported by the open nature of its design.

The main advantages of the solution presented in this study, discussed in detail in the previous paragraphs, can be summarized as follows: (i) integration of various state-of-the-art technologies and tools such as big data technologies and legacy technologies such as SQL databases; (ii) a 24/7 online operation by the deployment of cloud computing technologies; (iii) scalability and avoidance of technological entrapment due to the open source nature of all components; (iv) enhanced analysis and reporting capabilities that can be further expanded on demand by end-users and (v) the efficiency and robustness of the proposed solution as proven by its three years’ of continuous operation. 

Limitations and risks that were considered during the design of the DBDA solution were also mentioned in Section 2. Throughout the validation, testing and evaluation of the solution presented certain drawbacks and risks which were identified, pointing to the future research direction. First, appropriate training for end-users and stakeholders is needed, not only in view of learning the functionalities of the platform, but also in order to understand and familiarize themselves with big data technologies, analytics and privacy and security aspects, which would eventually enable them to fully exploit the potential of the platform developed. Moreover, the privacy and security aspects of the platform are of utmost importance, since access is mainly done in a remote fashion. Besides this, the cloud capabilities of the platform as well as the integration of big data technologies impose the need for constant consideration of cybersecurity aspects in order to avoid data leakage or any other malevolent actions that would compromise the platform itself. Another critical factor identified, as it could potentially lead to increased operational costs, is the employment of highly skilled IT personnel and data analysts in order to ensure smooth maintenance, update and upgrade of the technical components. 

Considering all of the above, prospects of the current solution envisage a constant training program through dedicated curricula for end-users, stakeholders, maintainer personnel and technical developers. The successful execution of such a training program, would really render the users able to fully exploit the potential of our solution. In addition, continuous development and roll-out of security and performance patches is required in order to support the system and provide a seamless operation, as the needs and the data volumes increase. Another likely future direction is the engagement of even more data providers, which could be partners operating different fleets or from different countries or locations, so as to expand the platform’s current functionalities and improve its results and performance. In the same direction, the enhancement of reporting and results capabilities of the platform will be further explored by engaging additional ML methods as well as more data and analysis results that will include prediction capabilities through semantic and reasoning.

## Figures and Tables

**Table 1:** The different data formats provided as input to DBDA platform from various sources.

Source	Variable	Unit	Description
Fleet 1	SpeedOBD	km/h	the speed measured by the OBD attached to the vehicle in km/h
	accuracy	number	the geolocation accuracy of the vehicle
	altitude	meters	the altitude from sea level measured in meters based on the vehicle’s geolocation
	bearing	degrees	a numerical value measured in degrees of the vehicle’s direction
	carPlate	string	a string of characters of the car plate
	engineRunTime	hours	a numerical value of the total time in hours which the engine of the vehicle was actively running
	engineTemp	°C	a numerical value of the current temperature of the engine at the time the measurements were recorded, in Celsius degrees
	fuelLevel	percentage	the percentage of the fuel that was in the tank of the vehicle at the moment the measurement was recorded
	fuelType	list	a numerical list indicating the type of fuel the vehicle uses
	intakeTemp	°C	the environmental temperature measured in Celsius degrees
	lat	degrees	the latitude of the vehicle location at the time the specific measurement was recorded
	lon	degrees	the longitude of the vehicle location at the time the specific measurement was recorded
	pendingTrouble	string	a string of characters indicating whether there were any alarming events or problems based on the diagnostics given by the OBD unit on the vehicle
	relThrottle	percentage	the percentage of the throttle’s position the moment the measurement was taken
	rpm	revolutions/min	the vehicle’s engine revolutions per minute
	speedGPS	km/h	the speed measured by the GPS of attached to the vehicle in km/h
	timeStamp	date and time	the date and time the measurement was taken in the format DDMMYYYY_HHMMSS
	vinNumber	string	a string of characters indicating the unique vehicle identifier given by the manufacturer
Fleet 2	Vehicle Name	string	a string of characters which indicates the license plate of each vehicle
	Vehicle ID	number	a unique number identifier for each vehicle
	DateTime	date and time	the date and time measurement in the format MM/DD/YYYY/HH:MM
	Day	date	the date measurement was taken in the format MM/DD/YYYY
	Raw Time	string	the time measurement in string format HH:MM:SS am/pm
	Time	time	the time measurement in time format HH:MM:SS am/pm
	lon	degrees	the longitude of the vehicle location at the time the specific measurement was recorded
	lat	degrees	the latitude of the vehicle location at the time the specific measurement was recorded
	Speed	km/h	a numerical value in km/h of the vehicle
	SpeedDiff	km/h	a numerical value of speed difference between two sequential time measurements in km/h
	TimeDiff	seconds	a numerical value of time difference between two sequential time moments in seconds
	Acceleration	number	a raw number value of acceleration
	AccelerationKm	km/h/s	The acceleration expressed in km per hour per second
Shifts	carplate	string	a string variable of the car plate
	date_from	date	the date measurement during the start of the shift taken in the format DD/MM/YYYY
	date_to	date	the date measurement during the end of the shift taken in the format DD/MM/YYYY
	km_start	km	the value of the vehicle’s odometer in km in the start of the shift
	km_stop	km	the value of the vehicle’s odometer in km in the end of the shift
	passenger_ID	number	a unique identifier of each driver
	shift_name	character	a single character which declares the time of the shift (M-morning, A-afternoon, N-night)
	time_from	time	the time measurement in the start of the shift in time format HH:MM
	time_to	time	the time measurement in the end of the shift in time format HH:MM

**Table 2:** The different data formats provided by the DBDA platform based on the analysis results.

Result	Variable	Unit	Description
Events	affected_users_count	number	the number of users that affected by an active event
	close_datetime	date and time	the date and time measurement during the end of an event taken in the format DD/MM/YYYY HH:MM:SS
	incident_id	number	a unique numerical identifier of the event
	incident_type	list	a list of numerical values for which each one indicates a different type of event
	lat	degrees	the latitude of the vehicle location at the time the specific measurement was recorded
	lon	degrees	the longitude of the vehicle location at the time the specific measurement was recorded
	origin	string	a string of characters declaring the origin of the event, e.g., driver’s mobile app or road administrator
	start_datetime	date and time	the date and time measurement during the start of an event taken in the format DD/MM/YYYY HH:MM:SS
	status	string	a string of characters declaring the status of the event, e.g., open/closed
	vinNumber	string	a string of characters indicating the unique vehicle identifier given by the manufacturer
Vehicle statistics	average_fuel	percentage	the average percentage of the fuel that was in the tank of the vehicle
	average_km_diff	km	the average difference in km of each vehicle based on the data of vehicles of fleet 1 and shifts
	average_relthr	percentage	the average percentage of the throttle’s position
	average_rpm	revolutions/min	the average of vehicle’s engine revolutions per minute
	average_speed	km/h	the average speed of the vehicle in km/h
	carPlate	string	a string of characters of the car plate
	max_fuel	percentage	the maximum percentage of the fuel that was in the tank of the vehicle
	max_km_diff	km	the maximum difference in km of each vehicle based on the data of vehicles of fleet 1 and shifts
	max_relthr	percentage	the maximum percentage of the throttle’s position
	max_rpm	revolutions/min	the maximum of vehicle’s engine revolutions per minute
	max_speed	km/h	the maximum speed of the vehicle in km/h
	min_fuel	percentage	the minimum percentage of the fuel that was in the tank of the vehicle
	min_km_diff	km	the minimum difference in km of each vehicle based on the data of vehicles of fleet 1 and shifts
	min_relthr	percentage	the minimum percentage of the throttle’s position
	min_rpm	revolutions/min	the minimum of vehicle’s engine revolutions per minute
	min_speed	km/h	the minimum speed of the vehicle in km/h
	records	number	a raw number of the total samples/records received from each vehicle
Driver statistics	average_fuel	percentage	the average percentage of the fuel that was in the tank of the driver
	average_km_diff	km	the average difference in km of each vehicle based on the data of drivers of fleet 1 and shifts
	average_relthr	percentage	the average percentage of the throttle’s position
	average_rpm	revolutions/min	the average of vehicle’s engine revolutions per minute per driver
	average_speed	km/h	the average speed of the vehicle in km/h matched to each driver
	driverid	string	a string of characters declaring the unique driver’s identifier
	max_fuel	percentage	the maximum percentage of the fuel that was in the tank of the vehicle per driver
	max_km_diff	km	the maximum difference in km of each driver based on the data of vehicles of fleet 1 and shifts
	max_relthr	percentage	the maximum percentage of the throttle’s position per driver
	max_rpm	revolutions/min	the maximum of vehicle’s engine revolutions per minute for each driver
	max_speed	km/h	the maximum speed of the vehicle in km/h for each driver
	min_fuel	percentage	the minimum percentage of the fuel that was in the tank of the vehicle per driver
	min_km_diff	km	the minimum difference in km of each driver based on the data of vehicles of fleet 1 and shifts
	min_relthr	percentage	the minimum percentage of the throttle’s position per driver
	min_rpm	revolutions/min	the minimum of vehicle’s engine revolutions per minute for each driver
	min_speed	km/h	the minimum speed of the vehicle in km/h for each driver
	records	number	a raw number of the total samples/records received from each driver
Driver’s behaviour labelling	average_rpm	revolutions/min	the average of vehicle’s engine revolutions per minute per driver
	average_speed	km/h	the average speed of the vehicle in km/h matched to each driver
	driverid	string	a string of characters declaring the unique driver’s identifier
	label	list	a predefined list with labels that each label corresponds to driver’s behaviour
	records	number	a raw number of the total samples/records received from each driver

## Data Availability

Not applicable.
